# A Robust Hybrid Approach Based on Estimation of Distribution Algorithm and Support Vector Machine for Hunting Candidate Disease Genes

**DOI:** 10.1155/2013/393570

**Published:** 2013-02-07

**Authors:** Li Li, Hongmei Chen, Chang Liu, Fang Wang, Fangfang Zhang, Lihua Bai, Yihan Chen, Luying Peng

**Affiliations:** ^1^Devision of Medical Genetics, Tongji University School of Medicine, Shanghai 200092, China; ^2^Key Lab for Basic Research in Cardiology, Ministry of Education, Tongji University, Shanghai 200092, China

## Abstract

Microarray data are high dimension with high noise ratio and relatively small sample size, which makes it a challenge to use microarray data to identify candidate disease genes. Here, we have presented a hybrid method that combines estimation of distribution algorithm with support vector machine for selection of key feature genes. We have benchmarked the method using the microarray data of both diffuse B cell lymphoma and colon cancer to demonstrate its performance for identifying key features from the profile data of high-dimension gene expression. The method was compared with a probabilistic model based on genetic algorithm and another hybrid method based on both genetics algorithm and support vector machine. The results showed that the proposed method provides new computational strategy for hunting candidate disease genes from the profile data of disease gene expression. The selected candidate disease genes may help to improve the diagnosis and treatment for diseases.

## 1. Introduction

Complex diseases are frequently accompanied by changes in gene expression patterns which can serve as secondary endpoints or biomarkers [1]. Microarray technology, which allows researchers to simultaneously measure expression levels of thousands or tens of thousands of genes in a single experiment, has been widely used to explore the gene expression pattern of complex diseases [2]. Typically, there are only a small number of genes associated with diseases. Thus, the selection of feature genes that possess discriminatory power for disease phenotypes is a common task for mining microarray data that are usually high dimension (with thousands of genes) and have small sample size (with usually a few dozens of samples) [3]. 

The method of gene selection generally falls into one of the following three categories: the filter, wrapper, and embedded approaches. The filter approach collects the intrinsic characteristics of genes in discriminating the targeted phenotype class and usually employs statistical methods, such as mutual information, statistical tests (*t*-test, *F*-test), and Wilcoxon's rank test, to directly select feature genes [4, 5]. This approach is easily implemented, but ignores the complex interaction between genes. The “wrapper” approach [6] aims at selecting a subset of feature genes, typically with an induction algorithm to search for an initial gene subset which can then be used for further evaluating new feature gene subsets. The wrapper method is usually superior to the filter one since it involves intercorrelation of individual genes in a multivariate manner. The wrapper method can automatically determine the optimal number of feature genes for a particular classifier. The embedded method is similar to the wrapper method, while multiple algorithms can be combined in the embedded method to perform feature subset selection [6, 7]. In the embedded method, genetic algorithms (GAs) [8, 9] are generally used as the search engine for feature subset, while other classification methods, such as KNN/GA (K nearest neighbors/genetic algorithms) [10], GA-SVM (genetic algorithms-support vector machine) [11], and so forth, are used to select feature subset. Estimation of distribution algorithm (EDA) [12] is a general framework of GA. Compared to traditional GA that employs crossover and mutation operators to create new population, EDA creates new populations by using a statistical approach to estimate the probability distribution of all promising individual solutions for the previous generation. EDA can also explicitly take into account specific interactions among the variables. When EDA is used to search for feature subsets, classification methods, such as Support vector machine (SVM) [13–19], which can deal with the high-dimension data in a limited sample space, can be used to select feature subsets.

In this study, we have developed a hybrid approach that combines both EDA and SVM (termed EDA-SVM) for selecting key feature genes. Here, EDA acts as the search engine, while SVM serves as the classifier, namely, the evaluator. We have applied EDA-SVM to two well-known microarray datasets: a colon data [20] and a diffuse large B cell lymphoma data [3]. Our results have shown that EDA-SVM can be used to identify a smaller number of informative genes with better accuracy in comparison to GA-SVM [11] and an estimation of distribution algorithm named PMBGA [21].

## 2. Materials and Methods

### 2.1. Description of DLBCL Datasets

We have applied the EDA-SVM method to the two following data sets: the diffuse large B cell lymphoma (DLBCL) data [3], available at http://llmpp.nih.gov/lymphoma/data.shtml, and the colon data [20], available at http://microarray.princeton.edu/oncology/affydata/index.html. The colon data set consists of 62 tissue samples including 40 tumors and 22 normal tissues, which cover 2000 human gene expression. The DLBCL data set harbors preprocessed expression profile of 4026 genes in tissues derived from 21 activated B-like DLBCL (AB-like DLBCL) samples and 21 germinal center B-like DLBCL (GCB-like DLBCL) samples.

### 2.2. Data Preprocessing

In DLBCL dataset, among 4026 genes, 6% genes have missing values and are imputed by the KNN Impute algorithm [22] prior to the EDA-SVM analysis. The KNN Impute algorithm uses the expression profiles of *K* nearest neighbors (here *K* = 5) to impute the missing values for the target gene. Therefore, in colon data *M*_0_ is a matrix with 62 rows and 2000 columns. In DLBCL data, *M*_0_  is a matrix with 42 rows and 4026 columns.

### 2.3. EDA-SVM

**Figure 1:**
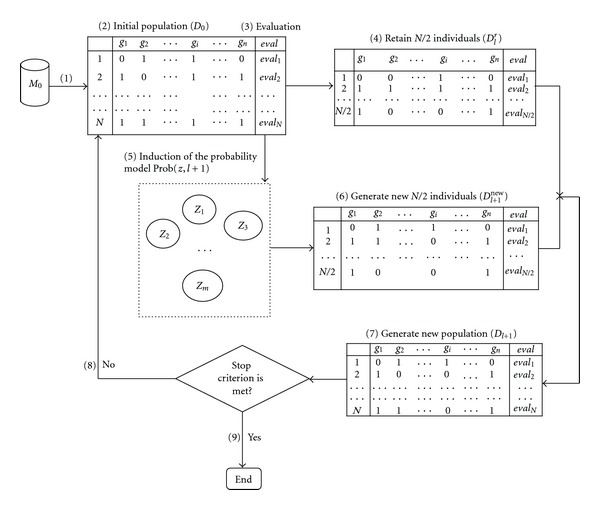
The main flow of EDA-SVM algorithm. *M*, *D*, *G*, and *eval* denote gene expression profile matrix, population, gene subset, and evaluation index, respectively.

Figure 1 shows the main flowchart of the EDA-SVM. EDA acts as the search engine, while SVM serves as the classifier, namely, the evaluator. The computational procedures are described in Algorithm 1. The major elements of the EDA include feature subset coding, population initialization, fitness computation, estimation probability distribution, generation of offspring and control of parameter assignment. At the beginning, we randomly generated the N fixed-length binary strings (individuals) to build up the initial population. Then, we calculated the fitness for each feature subset. Classification accuracy acted as the fitness index (fitness) that was evaluated using a linear SVM. The algorithm is an iterative process in which each successive generation is produced by estimating the probability distribution model of the selected individuals (parents) in the current generation and sampling the probability distribution to generate new offsprings. In this manner, reasonable subsets are developed successively until the terminal condition is fulfilled. In two data sets, lr is a learning rate and is assigned 0.08. Population size (*N*) is set as 40 and the maximal generations of 50 are determined, such that the solution space can be sufficiently searched while the best minimal subset can be obtained within the evolution time. 

 For each gene expression submatrix *M*_*j*_, we classify the microarray samples with genes contained in individual *j* using a linear SVM. The classifier, [18], is

(1) $$\begin{array}{c} \hat{y}=f\left(x\right)=\mathrm{sgn}\left(\sum_{i=1}^{L}a_{i}y_{i}K\left(x_{i}\cdot x\right)-b\right), \end{array}$$

then, the accuracy of classification is

(2) $$\begin{array}{c} \text{acc}=\frac{\left(\sum_{t=1}^{T}I\left(y_{t},\hat{y_{t}}\right)\right)}{T}, \end{array}$$

where *T* is the number of test samples and

(3) $$\begin{array}{c} I\left(y_{t},{\hat{y}}_{t}\right)=\{\begin{array}{cc} 1, & \text{if}\;\;y_{t}={\hat{y}}_{t},\\ 0, & \text{otherwise}. \end{array} \end{array}$$

The weight of each feature *i* in individual  *j* is

(4) $$\begin{array}{c} {\text{Pre}}_{\text{weight}}\left(z_{i}^{j}\right)=\{\begin{array}{cc} 0, & \text{if}\;\;z_{i}^{j}=0,\\ {\left(\sum_{h=1}^{L}\alpha_{h}y_{h}x_{h}\right)}^{2}, & \text{if}\;\;z_{i}^{j}\neq 0, \end{array} \end{array}$$

where *x* is a test sample vector and *x*_*i*_ is the learning sample vector. *L* is the number of learning samples. *y*_*i*_ is a class indicator (for a two-class application, +1 for the first class, −1 for the second class), and *a*_*i*_ is a nonnegative Lagrange multiplier associated with *x*_*i*_ and *a*_*i*_ ≠ 0 for support vectors. sgn( ) is the sign function and *K*(*x*_*i*_ · *x*) is the kernel function: linear kernel (*K*(*x*_*i*_ · *x*) = *x*_*i*_ · *x*, i.e., their inner product).

In this study, a fivefold cross-validation (CV) resampling approach is used to construct the learning and test sets. First, the two-class samples are randomly divided into 5 nonoverlapping subsets of roughly equal size, respectively. A random combination of the subsets for the two classes constitutes a test set, and the rest of subsets is totally used as the learning set. The 5-fold CV resampling produces 25 pairs of learning and test sets. Individual *j* is evaluated by the averaged value over the 25 pairs, that is,

(5) $$\begin{array}{c} \text{Fitnes}{\text{s}}_{j}=\frac{\left(\sum_{k=1}^{25}{\text{acc}}_{k}\right)}{25},\\ \text{weight}\left(z_{i}^{j}\right)=\frac{\left(\sum_{k=1}^{25}{\text{Pre}}_{\text{weight}k}\left(z_{i}^{j}\right)\right)}{25}, \end{array}$$

where *k* is the replicate number and acc_*k*_ is the classification accuracy for the *k*th replicate.

In the EDA-SVM algorithm, the optimization of the feature gene subset(s) is realized via survival competitions. For each generation, we retain 50% of the high-valued individuals that will directly enter next generation in order to keep these optimal solutions unchanged. On the other hand, in order to avoid the loss of the putative important feature genes, we initially contained about half of genes in each individual or preserving informative gene. Then, we adopt a stepwise data reduction procedure to minimize the feature subsets with more reliable classification accuracy. These gene expression matrices from the optimal individuals serve as the data on which the new round of iteration is performed. The data reduction process is completed once a stable gene subset is obtained.

### 2.4. GA-SVM

GA-SVM was previously developed [11] by us as a feature selection method. In GA-SVM, better feature subsets have a greater chance of being selected to form a new subset through crossover or mutation. Mutation changes some of the values (thus adding or deleting features) in a subset randomly. Crossover combines different features from a pair of subsets into a new subset. The algorithm is an iterative process in which each successive generation is produced by applying genetic operators to the members of the current generation. In this manner, good subsets are “evolved” over time until the stopping criteria are met. Thus, coding feature subset, population initialization, fitness computation, genetic operation, and control parameter assignment (population size, the maximal number of generations, and the selection probability) are the major elements of the GA-SVM method.

### 2.5. PMBGA

PMBGA can be applied for selection of a smaller size gene subset that would classify patient samples more accurately [21]. PMGBA generates initial population and builds a probability model and then selects individuals from the population. Probability distribution can be estimated based on the collection of selected individuals, and probability model can accordingly be amended so that a population is generated by sampling from the model. Instead of applying crossover and mutation operators in the process of generating new possible solutions (offspring), population can be updated in whole or in part relied on probability model. 

## 3. Results

### 3.1. Benchmark EDA-SVM

**Figure 2:**
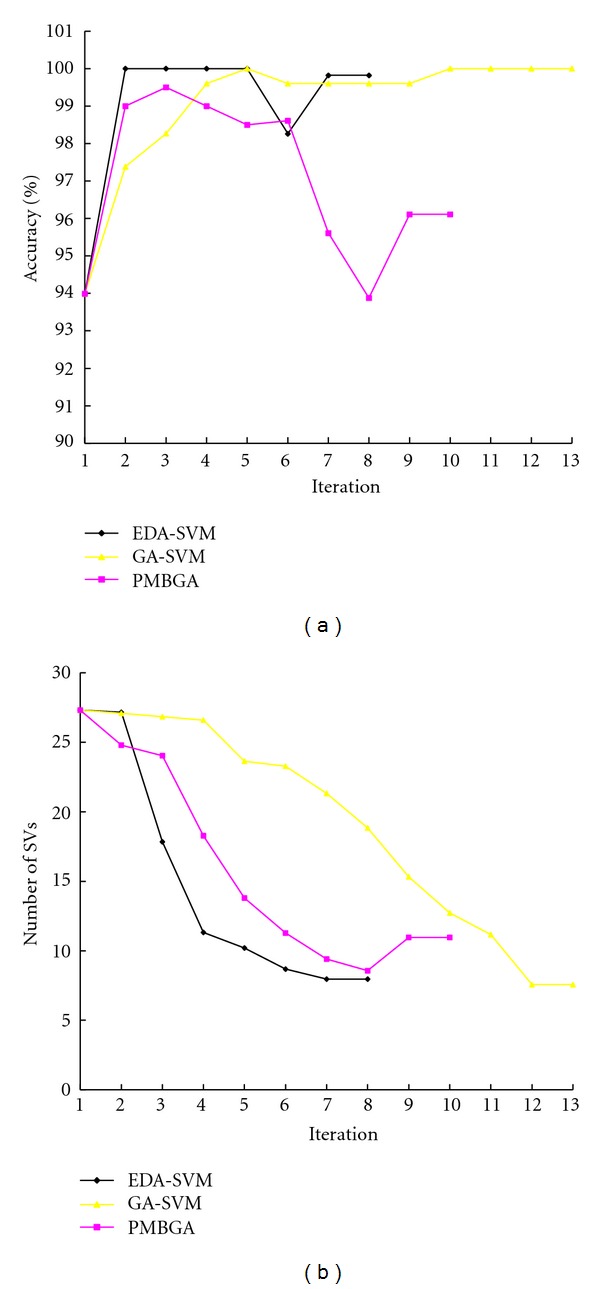
The changes of accuracy of the SVM classifier (a) and the changes of support vectors (b) over iterations in EDA-SVM, GA-SVM, and PMBGA based on DLBCL data set.

**Figure 3:**
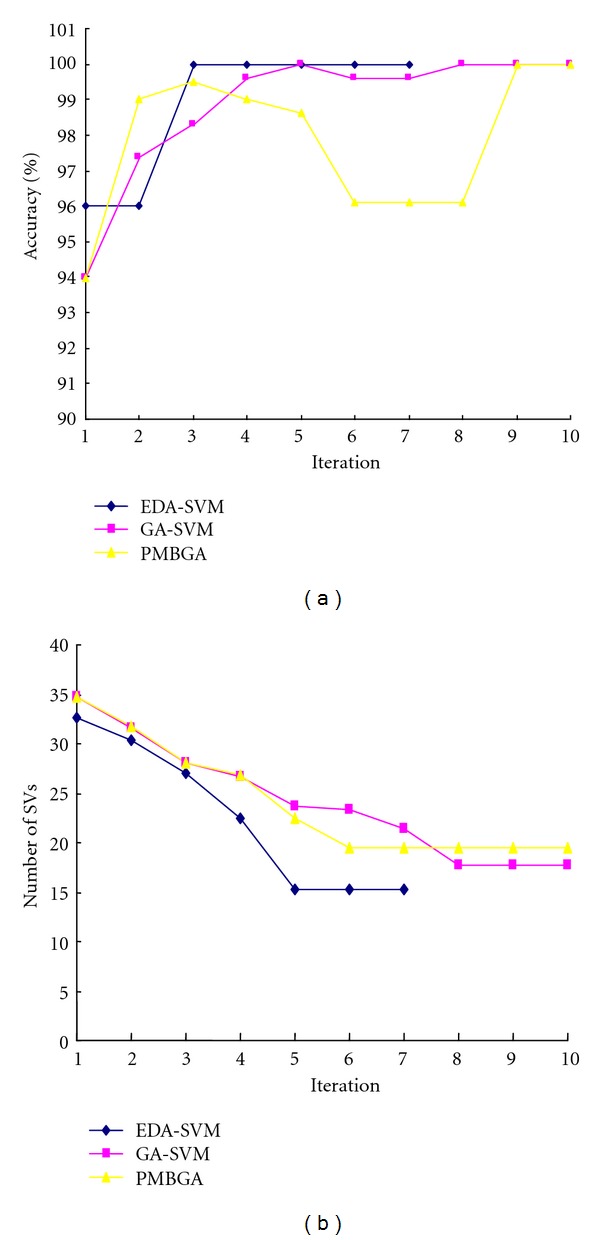
The changes of accuracy of the SVM classifier (a) and the changes of support vectors (b) over iterations in EDA-SVM, GA-SVM, and PMBGA based on colon data set.

The EDA-SVM method was applied firstly to the DLBCL data set. We started analysis with all 4026 genes and progressively reduced the dimension of the feature genes successively for 8 iterations after convergence. The accuracy of EDA-SVM increased from 0.9339 initially to 0.9982 at convergence (Figure 2(a)), while the number of feature genes at the successive generations is 4026, 460, 66, 17, 11, 7, 6, and 6, respectively (Figure 2(b)). For the colon data set, EDA-SVM reached accuracy of 1.0 after 7 iterations, and the final gene subset includes only 5 genes (Figure 3).

We compared the performance of EDA-SVM with two alternative methods: GA-SVM and PMBGA (Figures 2 and 3). The convergence speed of EDA-SVM is the fastest among the three methods. EDA-SVM converged after 8 and 7 iterations for the DLBCL and colon datasets, respectively. In contrast, it took 13 and 10 iterations for GA-SVM to converge, and 10 and 10 iterations for PMBGA to converge. Moreover, both the accuracy and the stability of EDA-SVM also show advantages among the three methods. EDA-SVM quickly reaches high accuracy after only a couple of iterations, while both the other two methods took more iteration to reach high accuracy. In addition, the accuracy of the other two methods had large variation during the iteration, while the accuracy of EDA-SVM kept stable during the iteration after it reached the high accuracy. 

### 3.2. Biological Analysis of the Selected Genes in the DLBCL Data

To understand the biological significance of the selected genes, we have analyzed the annotations of selected genes according to Gene Ontology (GO) (http://www.geneontology.org/) [23] and KEGG (http://www.genome.jp/kegg/kegg2.html) [24, 25] database. We selected six genes in the DLBCL data, which are SPIB, IRF8, NFKB2, LMO2, FCGRT, and BCL7B. The GO annotations of these six genes are shown in Table 1. Literature reviews of these six genes suggested that they are highly related to DLBCL. SPIB is an oncogene involved in the pathogenesis of AB-like DLBCL [26]. NFKB2 is a subunit of NF-*κ*B whose signaling pathway might contribute to the biological and clinical differences between the GCB-like and the AB-like DLBCL [27]. LMO2 was found to be located in the most frequent regime of chromosomal translocation in childhood T cell acute lymphoblastic leukemia. It was reported that LMO2 expressed at high level in germinal center B cell lymphocytes and at low level in AB-like DLBCL, respectively [3]. LMO2 is also one of the six genes in a multivariate model previously developed for prolonged survival in the diffusive large b-cell lymphoma [28]. BCL7B was found to be directly involved in a three-way gene translocation together with Myc and IgH in a Burkitt lymphoma cell line, and the disruption of the N-terminal region of BCL7B was thought to be related to the pathogenesis of a subset of high-grade B cell non-Hodgkin lymphoma [29]. BCL2 contributes to the pathogenesis in AB-like DLBCL [10] and is the common target gene of miR-21 and miR-221, both of which are overexpressed in AB-like than GCB-like cell lines [30]. Based on the above evidences, EDA-SVM successfully identified genes that may play role in the pathogenesis of DLBCL. 

## 4. Discussions and Conclusions

In this study, we have developed a hybrid method, EDA-SVM, which combines the estimation of distribution algorithms (EDA) with support vector machine (SVM) for selecting key feature genes from microarray data. Although similar combination strategies have been explored previously [21], EDA-SVM shows unique advantages compared with the alternative methods, GA-SVM or PMBGA. For example, EDAV-SVM not only converged more quickly, but also achieved higher accuracy with stable performance than the other two methods did. Both EDA-SVM and PMBGA [21] use EDA as the search engine, and SVM acts as evaluation classifier in feature selection procedure. However, there are several key differences between the two methods. First, EDA-SVM weights each feature using “*M*_weight_”, so that the contribution of each feature was fully considered during the update of each generation. In contrast, PMBGA assigns only a small random number to each feature. Second, for selecting minimal feature genes, EDA-SVM reduced the feature number step by step, while PMBGA did so by tuning the learning rate. Finally, the way to create the next generation in GA is also different between the two methods. As for the differences between EDA-SVM and GA-SVM, GA-SVM employs the traditional GA, while EDA-SVM generates new possible solutions (individuals) by sampling the probability distribution calculated from the selected solutions of previous generation. 

The structure of genes in a microarray data can be described by a Bayesian network. However, microarray data usually contains the expression of thousands or tens thousands of genes, making it virtually impossible to build a Bayesian network with so many genes. In this study, we have shown with EDA-SVM that proper combination of machine learning algorithms can overcome the high-dimension problem, and quickly converge to a small set of feature genes strongly related to target phenotype. The success of EDA-SVM thus made it readily applicable for hunting disease genes in microarray data. 

## Figures and Tables

**Algorithm 1:**
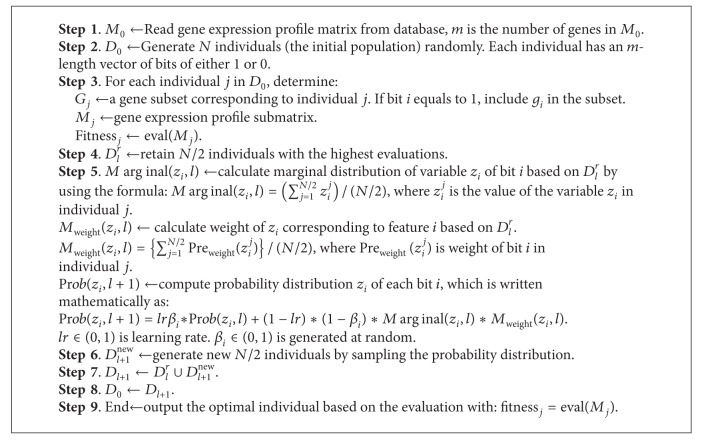
The step-by-step recipe for the computational algorithm of the EDA-SVM approach.

**Table 1:** The GO annotations of EDA-SVM feature genes.

Gene name Uigene ID	Biological process	Cellular component	Molecular function
SPIB (Hs.437905)	GO:0006350 Transcription	GO:0005634 Nucleus	GO:0003700 Transcription factor activity
	GO:0006357 Regulation of transcription from RNA polymerase II promoter	GO:0005737 Cytoplasm	GO:0003702: RNA polymerase II transcription factor activity

IRF8 (Hs.137427)	GO:0000122 Negative regulation of transcription from RNA polymerase II promoter	GO:0005634 Nucleus	GO:0003705: RNA polymerase II transcription factor activity, enhancer binding
	GO:0006355 Regulation of transcription, DNA-dependent		
	GO:0006350 Transcription		
	GO:0006955 Immune response		

NFKB2 (Hs.73090)	Go:0006355 Regulation of transcription, DNA-dependent	GO:0005634 Nucleus	GO:0005515 Protein binding
		GO:0005737 Cytoplasm	GO:0003713 Transcription coactivator activity
	GO:0007165 Signal transduction		GO:0003700 Transcription factor activity

LMO2 (Hs.34560)	GO:0008270 Development	GO:0005634 Nucleus	GO:0008270 Zinc ion binding
			GO:0005515 Protein binding
			GO:0046872 Metal ion binding

FCGRT (Hs.111903)	GO:0019882 Antigen presentation	GO:0042612 MHC class I protein complex	GO:0019864 IgG binding
	GO:0007565 Pregnancy	GO:0016021 Integral to membrane	GO:0004872 Receptor activity
	GO:0006955 Immune response		GO:0030106 MHC class I receptor activity

BCL7B (Hs.408219)	Unknown	Unknown	GO:0003779 Actin binding

## List of Abbreviations

DLBCL: Diffuse large B-cell lymphoma
EDA-SVM: Estimation for distribution algorithm-support vector machine
GO: GeneOntology
KEGG: Kyoto Encyclopedia of Genes and Genomes
GAs: Genetic algorithms
EDA: Estimation of distribution algorithm
AB-like DLBCL: Activated B-like DLBCL
GCB-like DLBCL: Germinal center B-like DLBCL
PMBGA: Probabilistic Model Building Genetic Algorithm
GA-SVM: Genetic algorithm-support vector machine.
